# A Practical Handbook of Diseases of Genito-Urinary System

**Published:** 1896-11

**Authors:** 


A PRACTICAL WORKING HANDBOOK IN THE DIAGNOSIS AND TREATMENT OF DISEASES OF THE GENITO-URINARY SYSTEM AND SYPHILIS. Based upon Clinical Lectures by Professor F. E. Doughty, of the New York Homœopathic Medical College, and edited by George Parker Holden, M. D. In press. Out soon.

